# Moving beyond ‘safety’ versus ‘autonomy’: a qualitative exploration of the ethics of using monitoring technologies in long-term dementia care

**DOI:** 10.1186/s12877-019-1155-6

**Published:** 2019-05-24

**Authors:** Alex Hall, Christine Brown Wilson, Emma Stanmore, Chris Todd

**Affiliations:** 1 School of Health Sciences, Faculty of Biology, Medicine & Health, University of Manchester and Manchester Academic Health Science Centre, Oxford Road, Manchester, UK; 2 School of Nursing & Midwifery, Queen’s University Belfast, Lisburn Road, Belfast, UK

**Keywords:** Ambulatory monitoring, Assistive technology, Dementia, Ethics, Implementation, Long-term care, Qualitative research, Surveillance

## Abstract

**Background:**

Use of monitoring technologies (e.g. wearable or environmental sensors) in long-term care generates extensive ethical debate, primarily about their potential to enhance resident safety weighed against concerns about their impacts upon resident autonomy. There are a number of other ethical aspects which are far less debated, including questions about the monitoring of the workforce, and equality of access to technologies. In this paper, we explore the extent to which remote monitoring of the workforce, and equality of access to technologies, were seen to influence the implementation of monitoring technologies within long-term care facilities.

**Methods:**

An embedded multiple-case study design was used with three dementia-specialist care facilities in England that had experience using a range of monitoring technologies. Data were collected through 175 h’ observation of daily practice, semi-structured interviews with 36 staff, residents and relatives, and examination of organisational documentation and technology manufacturer literature. Data were analysed using Framework Analysis.

**Results:**

Use of technologies for workforce monitoring was understood in relation to the ethical obligations to fulfil a duty of care to residents. There was little recognition of any negative implications for the workforce, but staff were susceptible to rumours that technologies were being used for performance management even when this was not the case. There were questions about how far data collected by monitoring technologies could constitute ‘evidence’ of appropriate care delivery. Equality and access to technologies involved a need to compromise between generic designs that were not universally suitable, but were more affordable than bespoke designs. Contracts with suppliers imposed limitations on product choice.

**Conclusions:**

There is an urgent need for greater consideration of the ethical and legal implications that remote technological monitoring might have upon workforce morale, recruitment and retention. Ensuring variety of technological design to facilitate equitable access for residents is financially extremely challenging. It is possible that considerations of equitable access are not deemed a priority due to the current generation of residents’ low levels of technological familiarity and expectation. It might be overstated and unrealistic to view expensive technologies as the pinnacle of innovative practice in care homes.

**Electronic supplementary material:**

The online version of this article (10.1186/s12877-019-1155-6) contains supplementary material, which is available to authorized users.

## Background

There is increasing interest in technologies to support people with dementia to live as well as possible with a condition for which there is no imminent cure [1, 2]. Technologies for dementia support, often labelled ‘assistive technologies’, may be grouped into three overlapping categories: devices used ‘by’ people with dementia, e.g. for prompts and reminders; devices used ‘with’ people with dementia, e.g. to support communication and reminiscence; and devices used ‘on’ people with dementia, e.g. to monitor activity, movement and location [3]. The progressive nature of dementia means that many people ultimately require long-term residential or nursing care; hereafter referred to as ‘care homes’ [4]. Care homes tend to favour technologies used ‘on’ residents, primarily to enhance safety and help fulfil duties of care [5]. These technologies include integrated systems that incorporate wearable and/or environmental sensors to detect bed occupancy, falls, entry and exit through doorways, and provide continuous monitoring of location, activity, and/or physiological signs; all of which may alert staff when assistance is required, and generate data for post-hoc analysis. There is also growing interest in video technology [6, 7], largely to tackle fears of malpractice, but also to illuminate unwitnessed incidents such as altercations between residents [8]. All these technologies are hereafter referred to as ‘monitoring’ technologies.

There is extensive ethical debate surrounding the use of monitoring technologies in dementia care, much of which draws upon the apparent conflict between the biomedical ethics principles of autonomy (respect for independence, freedom, privacy), weighted against beneficence (do good) or non-maleficence (do no harm) [9]. This conflict is an enduring, generic challenge for care home staff, since any acts of care may intrinsically involve some level of interference with resident autonomy [10]. Use of monitoring technologies amplifies this conflict, and there is a lack of guidance on how to manage such conflict [11]. There is some recognition amongst care home staff and relatives that monitoring technologies may be able to protect residents from harm whilst affording them more autonomy [12–17]. However, primacy tends to be placed on the potential of these technologies to support staff to uphold their duty of care through harm prevention, and issues such as resident independence, freedom and privacy are often less considered, or deemed less important [13] [17–20]. There are also challenges in assimilating models such as the biomedical ethics principles with broader societal standards of morality [21], and in considering how the biomedical principles relate to other frameworks such as care ethics which advocate empathetic relationships between residents and staff and give priority to the immediate moment [22]. Care ethics underpin concerns that monitoring technologies might replace staff-resident contact and might be antithetical to person-centred care [5]. However, monitoring technologies may help staff direct their attention toward residents who need support in the moment, and thus could be seen to facilitate relevant and timely care, which is a mark of compassion and a person-centred way of working [12, 13, 23, 24].

Niemeijer et al. [5] concluded their comprehensive review of monitoring technologies by stating that there is no consensus on their ethical viability in residential dementia care*.* In a systematic review to develop a framework of categories of disease-specific ethical issues for dementia care, Strech et al. [25] categorised use of monitoring technologies as a special situation for decision making (alongside other situations such as antipsychotic drugs, sexual relationships, and ability to drive), highlighting how use of monitoring technologies may extend beyond other categories of ethical issues such as ‘decision making and consent’ that are too narrow to account for the complexity of the situation. These positions are unsurprising because they are contingent upon diverse ethics values, frameworks, and codes of conduct, which may be challenging for health and care professionals to apply in daily practice [26]. The ongoing debate around the relationship that monitoring technologies have with duty of care, autonomy, and person-centred care exemplifies the observation that good dementia care does not have a simple hierarchy of values [27], but rather, requires ongoing assessment tailored to the continually changing needs of individuals. The use of monitoring technologies also presents a number of complex legal considerations for care providers, including (but not limited to) data protection and human rights legislation. For example, in the UK, the health and social care regulator offers detailed information to guide consideration of the use of monitoring technologies, but repeatedly recommends seeking legal advice [6].

There are at least two other pertinent areas of the ethical debate that are far less discussed. The first area is the potential for the remote technological monitoring of the care home workforce. This occurs ever-more frequently in the general workplace, with scant regard for ethical issues [28]. It is important to acknowledge that care homes have multiple identities, functioning simultaneously as people’s homes, as sites of health care, and as workplaces; this multiplicity is likely to present a range of perspectives regarding the role of monitoring technologies that are extremely difficult (if not impossible) to reconcile. Most debate about workforce monitoring has taken place in the USA, where some states have legalised the use of monitoring technologies with laws that ostensibly protect the privacy of the workforce via visual notification (e.g. signage) that monitoring is occurring [29]. In theory, workers can modify their behaviour in a space that they know is being monitored, or can protect their privacy by removing themselves from such spaces, but in reality they seldom have these choices as they have to provide care for residents wherever it is required [29]. There is therefore an underlying assumption that monitoring is used as a tool against workers rather than in a supportive way [29]. The second area is equality of access to technologies, at both the micro level between individual residents of a care home, and at the meso level between care homes within the social care sector [5]. International funding models for social care are diverse and complex, but many incorporate elements of public and private funding [30]. In England, the cost of residential care is means-tested, with a relatively high threshold of eligibility; the cost of nursing care is assessed on clinical need and is partly or wholly funded by the National Health Service (NHS) [31]. This means that there may be a mixed funding model operating both throughout the sector and within individual care homes. Variability in price and affordability of monitoring technologies raises concerns of two-tier access [2], particularly in a sector under sustained financial pressure [32]. In their review of ethics of using assistive technology with community-dwelling older adults, Zwijsen et al. [33] discussed how large-scale implementation of technology could lower costs and help to tackle the problem of equity, but highlight that the trade-off of such a collective approach may be to obscure consideration of other ethical issues at an individual level.

We aim to contribute to these two lesser-explored areas of the ethical debate. In the present paper, we draw upon data from a study that explored the implementation of monitoring technologies in care homes. One of the objectives of this study was to explore the influence of the ethical debate upon the implementation of monitoring technologies in care homes. In the present paper we explore the extent to which remote monitoring of care home staff, and equality of access to technologies, were seen to influence the use of monitoring technologies within routine practice, and discuss subsequent ethical implications.

## Methods

### Design, settings, and technologies

In their seminal review of implementation of interventions in health service organisations, Greenhalgh et al. [34] recommended that implementation research should explore how causes and effects happen rather than simply whether an intervention ‘works’, and pay attention to the context in which implementation takes place. These recommendations point to the appropriateness of case study as a methodological approach because it affords practical, context-dependent knowledge which remains true to the complexity of the context studied [35]. Yin [36] outlines a variety of case study designs consisting of a context, in which sits the case, containing units of analysis. We adopted an embedded multiple-case study design [36], in which we defined the context as a particular care home, containing the case defined as the process of implementation of monitoring technologies. The ‘embedded’ aspect of the design refers to distinct units of analysis within each case; we identified these as the perspectives of staff, residents and relatives within the home, plus organisational documentation and technology manufacturer literature.

We recruited three dementia-specialist care homes in urban areas of Northern England. For confidentiality purposes, we renamed these homes Sycamore Lane, Conifer Gardens, and Heather Grove. Local research networks were used to aid recruitment, guided by a purposive approach, as the homes differed in size, care provision, technologies used, and ownership structure. Sycamore Lane and Conifer Gardens were purpose-built homes providing residential care with nursing. Heather Grove was a converted older building, providing residential care without nursing. Sycamore Lane and Heather Grove were each part of (different) for-profit local chains; Conifer Gardens was part of a not-for-profit national chain. Each home was using a nurse-call system that incorporated bed-exit monitoring and staff alert capabilities. Each home also had experience with other monitoring technologies, including a wearable activity tracker (Sycamore Lane), wearable location-tracking technology (Conifer Gardens), and door monitoring technology (Heather Grove). Table 1 provides further detail about the technologies, including types, appearance, location, functionality and methods of operation.

**Table 1:** Descriptions of technologies in each care home

Technology	Care home	Technology type, appearance and location	Functionality and method
Nurse Call	Sycamore Lane	System comprised of nurse call buttons, bed sensors installed underneath mattresses, pagers carried by staff to which all alerts are sent, and central computer which records data about alerts and resident vital signs. Bed sensors plug via wire into units affixed to headboards, which also contain green, red and blue buttons for staff to record attendance, call for help or an emergency alert respectively. Bed sensors and units can be moved between bedrooms (and would require recoding via central computer to update assigned room number). Nurse call buttons in communal areas can be detached and moved to different locations. All units and nurse call buttons wirelessly transmit to central computer and to pagers.	Requires touch or pressure from user (active or passive). Allows communication between user (resident or staff) and assistance (staff). Bed sensors can record continuous observation of vital signs. Bed sensor activates upon movement; non-movement e.g. seizure; can be set to timed delay to account for mobility level of resident. Nurse call buttons can be pushed.
Nurse Call	Conifer Gardens & Heather Grove	System comprised of nurse call buttons and pull chords, pressure mats, wall units to which alerts are sent, and central computer which records data about times of and response times to alerts. Wall units can be used to record attendance, call for help, or generate emergency alert. Pressure mats can be moved within bedroom e.g. placed by bed or in front of chair, but cannot be moved from bedroom due to wired connection to socket installed in bedroom wall. Wall units hardwired into walls and cannot be moved.	Requires touch or pressure from user (active or passive). Allows communication between user (resident or staff) and assistance (staff). Pressure mat activates upon contact. Nurse call buttons and chords can be pushed/pulled.
RFID location-based System	Conifer Gardens	System comprised of fobs, sensors, pagers, and central computer. Individual fobs worn by residents; pagers carried by staff; sensors installed in ceiling. Records information about resident mobility, including steps, location, and duration of activity. Also capable of recording information about staff location and activity via monitoring of pagers. Data accessible from central computer. Radio-Frequency Identification (RFID) allows for assignation of unique person identifiers and allows personalisation of system, e.g. bespoke alert parameters according to resident level of need.	Automatically detects location of fobs and pagers via RFID. Allows communication between user (resident or staff) and assistance (staff). Continuous observation of fobs and pagers.
Activity Tracker	Sycamore Lane	Accelerometer clipped to clothing or carried in pocket. Records information about resident’s mobility, including steps, duration of activity, distance and caloric burn. Data logged in ‘cloud’, accessible online.	Continuous monitoring of user activity, gathers data arising from movement.
Door Monitors	Heather Grove	Magnetic sensor wireless tags approximately 25cm^2^ affixed to bedroom doors. Record information about time and duration of door opening. Data logged in ‘cloud’, accessible from laptop in manager office.	Require movement of door to activate; automatic recording of time and duration of opening.

### Participants, recruitment, and consent

Staff members, residents and their relatives were eligible for inclusion if they were over 18, could speak English, and had any involvement with monitoring technologies. The first author (AH) was responsible for participant recruitment and data collection. He had no prior knowledge of or relationships with people within each home, and therefore at the outset of the study he undertook a period of acclimatisation, introducing himself to staff, residents and relatives, and explaining the study. After this, participants were identified and invited to take part, either directly by AH or with the help of key staff members. Sampling was purposive, including staff with varying roles and shift patterns, and residents with different levels of cognitive impairment and care needs. Participants were provided with written information, offered the chance to discuss the study with AH, and gave written consent.

AH sought guidance from staff and family members about the capacity of residents to consent to participation, and for the vast majority it was advised that they lacked capacity to give their informed written consent. In these situations, we followed Dewing’s [37] process model of consent. This is a stepped model which first involves approaching a consultee (usually a relative) to ascertain whether a resident would potentially be interested in taking part. The consultee was given an information sheet about the study, which also outlined the role of consultee and why they were being approached, and explained why the person with dementia was being invited to participate. The consultee was asked to consider this information from the perspective of the person with dementia, and asked to sign a declaration form stating that in their opinion, the person with dementia would have no objection to taking part in the study. This declaration form contained a very similar list of items as a standard consent form - i.e. that participation was voluntary and that the person could be withdrawn if they did not wish to continue participating, that the researchers may take notes/observational field notes, and that in their opinion, the person would consent to use of anonymised quotes. The process model also involves the researcher learning about the resident’s communicative behaviours, and monitoring each interaction with the resident for signs of distress. In this model, the consultee thus grants initial permission for the researcher to approach the resident, and the researcher then uses detailed knowledge of the individual resident’s behaviours and communication to judge whether or not that individual is giving consent. In most cases, the mechanism through which consent is given is not through the traditional written method. Instead, it requires detailed recording and reflection from the researcher about each interaction they have with the individual, based on what they know about that individual’s behaviours and communication style, and discussion and feedback with members of staff and relatives in order to monitor consent on an ongoing basis. It places the person with cognitive impairment at the heart of the consent process, emphasising relational aspects of care, and allows for the inclusion of participants who are often excluded from research [37].

### Data collection, analysis, and quality assurance

Data were collected between February and November 2014, using primarily non-participant observations, and semi-structured interviews with staff and relatives. We attempted to interview residents, but owing to levels of cognitive impairment, in the main we used informal conversation during observations. Observations were overt, and included all aspects of day-to-day activity except for personal care. Observation notes were recorded by hand. Interviews were held in quiet areas of the homes, at times which suited the participants. The vast majority of interviews were audio-recorded and transcribed verbatim; a minority were recorded by hand if it was not possible to conduct the interview in a quiet place. We also collected data pertaining to technology use from care home records, and technology manufacturer literature (product websites and operational guidance).

All data were collected by AH (male, PhD candidate with experience of social care practice and research). He regularly discussed data collection with CBW, ES (both female, registered nurses experienced in dementia care practice and research) and CT (male, chartered psychologist experienced in the development of technologies for falls prevention). We conducted 175 h of observation, and interviewed staff from all levels of the organisational hierarchies, including managers, clinicians and support workers (*n* = 24), relatives (*n* = 9), and residents (*n* = 3). Two relatives declined to participate, and declined approaches to their family member residents, on the grounds that participation would be an unwanted burden. We extracted data from nine residents’ care records (relating to technology use only, not personal medical histories), five product websites, and one product training manual. The mean ages of participants were 39.75 years (staff; range 21–64); 55.67 years (relatives; range 41–78); and 81.33 years (residents; range 72–95). Most participants had been involved with the homes for around 2 years, either as staff members or as residents and relatives, although many staff members had prior career history within social care. The vast majority of participants were female, of White British ethnic origin. All participants were given pseudonyms, and any other identifying information, such as place names, were removed prior to analysis.

Data collection was informed by Normalization Process Theory [38], to guide focus upon participants’ understandings, levels of involvement, uses-in-action, and evaluations of technological interventions as they attempt to make them part of routine practice. The interview topic guide is provided as in Additional file 1. The interview guide was not pilot tested, but was used as an heuristic device to help the interviewer ensure that interviews covered the range of issues highlighted by Normalization Process Theory. The precise wording of the questions varied during data collection to suit the individual participant. Interviews lasted 22–90 min with staff (most often about 40 min) and 16–35 min with relatives (most often about 25 min). Data were collected iteratively, so many interviews contained additional questions to triangulate observations or points raised by participants in earlier interviews.

We analysed the data using Framework Analysis [39], supported by NVivo 10 software. We followed the systematic approach inherent to Framework Analysis, beginning with familiarisation with data and the development of a working framework, moving through refinement of the framework via further coding and explicit charting of data, ending in final interpretation of the major themes [40]. Framework Analysis is one of a range of methods within the broad church of thematic approaches to analysis; its unique feature is a matrix output which allows data to be compared and contrasted across and within cases [40]. This makes it particularly well-suited to the systematic approach advocated by Yin [36] for cross-case synthesis of multiple-case study designs.

Analysis was led by AH, supported through regular consultation with CBW, ES and CT. Data collection continued until data saturation – i.e. no new findings emerged during analysis [41]. Interview transcripts were not returned to participants; Hagens et al. [42] have argued that spontaneous responses may be of more value than responses that have been modified through member-checking, and also found that there may be extremely low numbers of participants who take up invitations to review full transcripts. Based on our experiences in the study of recruiting very busy participants, we decided not to place further burden upon them by giving them additional reading material. No participants requested to see transcripts. Data collection was iterative therefore a degree of member-checking was employed as participants were invited to provide comment upon our emerging analysis. Our triangulation of multiple methods of data collection, transparent analysis approach, and clear methods description are markers of quality in qualitative research [43].

### Ethics approval

Ethics approval was granted by the NHS National Research Ethics Service Committee North West – Haydock (reference 13/NW/0752).

## Results

**Fig. 1:**
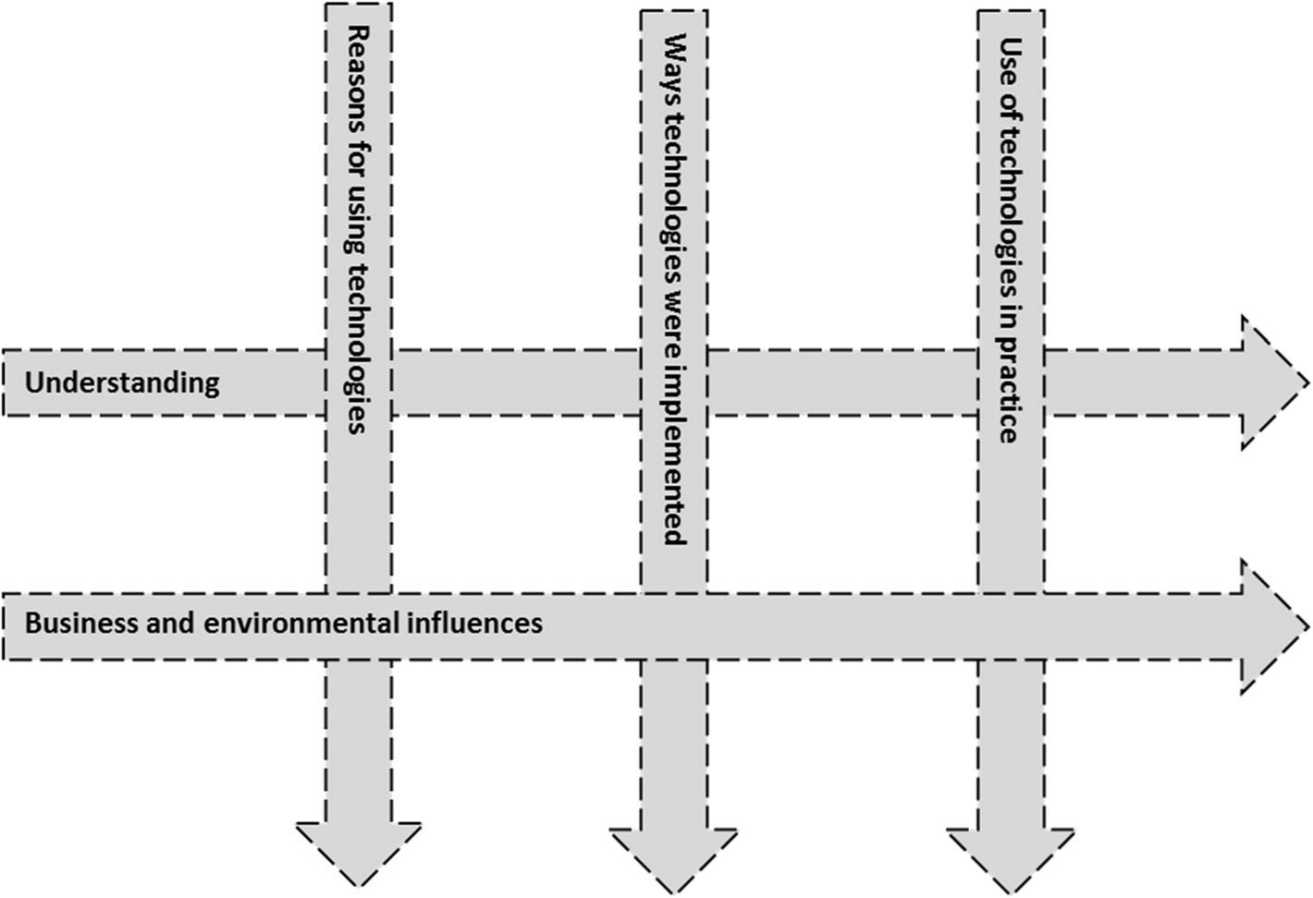
Analysis matrix of main themes

Analysis resulted in a final coding framework of 49 codes grouped into five themes: (i) Understanding; (ii) Business and environmental influences; (iii) Reasons for using technologies; (iv) Ways technologies were implemented; (v) Use of technologies in practice. These themes were arranged in a matrix, shown in Fig. 1.

We present findings according to the two horizontal themes in Fig. 1, ‘Understanding’ and ‘Business and environmental influences’. The broken lines in Fig. 1 represent the permeability of thematic boundaries; in other words, ‘Understanding’ and ‘Business and environmental influences’ were each developed in reciprocal relationship with aspects of ‘Reasons for using technologies’, the ‘Ways technologies were implemented’ in practice, and experiences of ‘Use of technologies in practice’.

### Understanding

This theme primarily captured participants’ frames of understanding around the use of monitoring technologies, including the influence of societal narratives and stereotypes. In interviews, participants all understood monitoring technologies as existing fundamentally to enhance safety. This understanding was also documented in resident care plans, for example, bed-monitoring technology use was recorded as part of a falls prevention or reduction strategy for individual residents. None of the homes was using video technology, but several participants discussed the idea of using video technology to enhance safety further, including through workforce monitoring. One relative at Sycamore Lane suggested that cameras would *“keep everyone on their toes”* (Alice, daughter, Sycamore Lane). Some staff members appeared to be tentatively in favour of video cameras, suggesting that they would encourage transparency, shaped by understandings that *“if you have good practice, you’ve nothing to hide”* (Ernie, care worker, Sycamore Lane). However, these positions were not universally advanced. The managers at Conifer Gardens were the most uncomfortable with the idea of video technology, albeit primarily on the grounds of invasion of resident privacy rather than any impact it might have upon staff. They advocated alternative approaches to workforce monitoring grounded in organisational policies and culture:

*“there must be, there has to be [alternatives to video cameras]… good whistleblowing policies… making sure your staff are open and honest, making sure your staff will not accept anything other than the very best… the threshold is if [care] is not good enough for your own family, it isn’t good enough for the people who live here”* (Ben, deputy manager, Conifer Gardens)

The managers at Heather Grove had implemented the door monitors specifically to monitor night staff activity as they were concerned about what happened when they themselves were not in the building:

*“we shouldn’t be stereotypical cos you’re supposed to trust people you work with, but the [night staff] sleep a lot don’t they”* (Kathy, deputy manager, Heather Grove)

The deputy manager stated that this understanding had been influenced by stereotypical images of care homes, reinforced by recent UK media depictions of scandals in care homes. The manager in this study reported that this care home had deliberately not informed night staff about the door monitors in advance of installation. She believed staff had little choice but to accept the technology, since it only served to compel them to carry out the regular checks on residents that were required of their role. One member of the night staff suggested that she had eventually accepted the technology, since she understood that the managers held ultimate accountability for the safety of the residents, but felt that she should have been informed about the technology in advance of its use (Natalie, night care worker, Heather Grove).

Findings from the other two homes demonstrated that some staff believed that they might be monitored by technology, even when this was not the case. At Sycamore Lane, staff expressed such concerns about the activity tracker, including the manager, who reported that she had initially thought the home’s owner was *“trying to track me down”* when he had left a tracker for her to test out (Erica, manager, Sycamore Lane). At Conifer Gardens, some night staff felt that the RFID system had been used as a *“Big Brother tool for management”* to monitor staff activity (Judy, night care worker, Conifer Gardens), although senior staff suggested that this had not been the case. The training manual for the RFID system explicitly cautioned that using the system to discipline staff would *“undermine the value of the technology”*.

The deputy manager at Heather Grove stated that prospective families were shown the door monitors, pointing to the potential for reassurance that the home was holding its staff accountable:

*“when anyone shows people round, we point out the [door monitors]… it sort of puts people at ease... if someone was ever concerned… you can show them [the data that staff have checked on a resident]”* (Kathy, deputy manager, Heather Grove)

At Conifer Gardens, there was similar support for this potential use of the RFID system:

*“if you have an anxious family, [you could] say there’s the empirical data to say [the resident was] checked at this time, this time, this time, this time and this time”* (Harry, clinical lead, Conifer Gardens)

At all three homes, alerts and response to alerts from the nurse call systems were logged by central computers. These data could similarly be used to defend the home against allegations of negligence, which had happened at Conifer Gardens:

*“there was an allegation a while back that [an alert] wasn’t answered for 20 minutes... I was able to go into the system and see that it was answered within four minutes”* (Ben, deputy manager, Conifer Gardens)

However, the strength of such data as evidence that appropriate care had been delivered diminished under scrutiny. One care worker reflected on her experience of working in a different home, which had set staff time targets for responding to alerts. She reported that if staff were delivering care to a resident and another alert was unanswered, they would temporarily excuse themselves from that resident to turn off the alert in the other room, before returning (Natalie, night care worker, Heather Grove). This suggested that there may have been a difference between responding to an alert and actually delivering care to the specific resident. There was similar recognition at Conifer Gardens that data gathered from the RFID system about staff activity would not necessarily prove that care had been delivered:

*“if pressure relief’s being done in bed, as in turning the person, it doesn’t necessarily mean that you’ve turned the person if you’ve gone into the room, it just means you’ve gone into the room”* (Harry, clinical lead, Conifer Gardens)

This example of pressure relief was hypothetical, but in general, the managers suggested that turning to technology to address any such problems would be misguided. They felt that solutions to would be found in *“[understanding] what are the hidden causes”*, rooted more deeply in staff attitudes and organisational culture, rather than *“buying a piece of kit to fix the issue”* (Philippa, manager, Conifer Gardens).

In summary, understandings of monitoring technologies included considerations of their use for monitoring the workforce, either for management to hold staff to account and uphold standards of care, or to defend the home against accusations of malpractice. However, there were questions about how far data collected by monitoring technologies constituted evidence that appropriate care had been delivered, and whether this type of monitoring was preferable to broader, robust organisational policies and a culture of openness. From an implementation perspective, remote monitoring of the workforce was therefore understood according to its potential utility relating to the ethical obligations inherent to a duty of care to residents. Ethical objections seemed to be made in relation to the potential impact upon residents (i.e. of video technology), with little overt understanding of any ethical implications of workforce monitoring for the staff members themselves.

### Business and environmental influences

This theme primarily captured the influence of business considerations and environmental factors upon the use of monitoring technologies in practice. All three homes contained a mixture of private- and publicly-funded residents. The provision of some technologies, such as specialist wheelchairs, was arranged by individual residents and their families, which suggested that more affluent families would have preferential access to these types of assistive technologies. There were sources of funding support available for these technologies (e.g. local authority grants), but such support required families to make a specific application to the funder. In contrast, all monitoring technologies were paid for by the care homes, and thus there was no evidence that differences in funding sources influenced unequal access to monitoring technologies. However, there were signs of a potential challenge for care homes in managing distribution of communal resources against individual family demands. At Conifer Gardens, there was an example of one resident who had kept falling out of bed, but whose family refused to allow the bed to be placed against a wall to protect one side. The home had therefore used two pressure mats with this resident, one on either side of the bed. One care worker pointed to the resource implications of this distribution, suggesting that *“in most cases you wouldn’t have two mats, cos they are worth quite a bit of money”* (Simone, care worker, Conifer Gardens). A relative, talking about resource distribution in general, stated *“you can’t take one off someone else and put it with someone else”* (Colin, son, Conifer Gardens).

Some staff members reflected that the design of technologies, including pressure mats and the RFID fobs worn by residents, might be temporarily or permanently unsuitable for residents who lacked capacity to understand the technologies and their component parts. One nurse argued that the technologies were incompatible with truly person-centred care, but recognised the financial barriers to bespoke design:

*“it should be person-centred, and technology isn’t person-centred… you’ve got the technology, but you can’t use it until he’s capable of accepting [it]… you can’t treat everybody the same, and that’s where technology falls down, because it’d be too [expensive] to personalise it, and then who’d pay for that?”* (Olivia, nurse, Conifer Gardens)

Problems with obtaining a wider range of products were not solely influenced by unit cost. For Conifer Gardens, as one home within a larger parent company, the availability of technologies was partly restricted by organisational procurement strategy and contractual obligations:

*“we’ve got to fall in line with an overall strategic position within our organisation with certain contracts and certain available products, so sometimes we’ve got to make what’s available to us try and best fit the people that we support… it's a lot of work to do a full [new] business case for one product [not on the approved list] that might only be £150, when we can order a pressure mat [already approved] for £80 no questions asked”* (Ben, deputy manager, Conifer Gardens)

There was also a preference for components made by different manufacturers to have interoperability, so that managers could *“do your homework and shop around”* (Philippa, Manager, Conifer Gardens), but often this was not facilitated by manufacturers. At Sycamore Lane, nurse call system buttons in communal areas could be moved around the building, but this work seemed to require a charged call-out of the system manufacturer. The facilities manager felt it was unnecessary *“to pay a guy £150 for a day to drill three small holes in a wall and attach a wireless device”* when he seemed to be able to do this work himself (Noel, facilities manager, Sycamore Lane). These findings suggested that manufacturer exclusivity of products and maintenance may be financially costly for care homes, which in turn could preclude certain residents from accessing variations of monitoring technologies that might be more suitable for their needs.

Conifer Gardens was arranged into separate households, providing residential or nursing care. The sensors for the RFID system had been installed on one residential care household on the first floor, and in the ground floor reception area. If residents wearing fobs walked out range of the sensors, an alert was repeatedly activated. This problem of a false alert could have potentially been rectified if the system had been installed throughout the entire building, but the cost of doing so had been prohibitive:

*“in all honesty it was a cost thing, our plan was to spread it over to the other side of residential and down into the garden”* (Harry, clinical lead, Conifer Gardens)

The manager reflected on challenges of relying on technology for innovation, and suggested that there was better value to be found from other forms of innovative practice:

*“people pay £[x] to live here, if they’re private, most of the people that live here come through as local authority [funded], so we’re getting [less than £x] a week... we're looking for innovation but [the RFID system] is a really costly piece of kit… we don’t charge [name of affluent locality] rates... so our passion is looking for innovation through people”* (Philippa, manager, Conifer Gardens)

Both the owner at Sycamore Lane, and the managers at Conifer Gardens, expressed scepticism toward technology manufacturers who justify charging care homes high prices for their products by invoking high quality and safety. Costly technologies at times lacked the robustness necessary to withstand the demands of daily practice:

*“you’ve got seven staff on a day shift, so you should have seven pagers, that’s two grand [£2000], two and half grand’s [£2500] worth of kit just attached to someone’s hip, that gets put in the bath, goes down toilets, get smashed”* (Ben, deputy manager, Conifer Gardens)

At Sycamore Lane, some staff seemed reluctant to take on the responsibility of a pager because of the cost. There was a rumour circulating amongst staff that they would be held personally responsible for the cost of replacing damaged pagers. However, senior staff suggested this rumour was unfounded:

*“the organisation replaces it… it’s cost a fortune in the last two years for pagers”* (Tiffany, head nurse, Sycamore Lane)

George, the Sycamore Lane resident using the activity tracker, reported that he had.

lost three trackers, and that they cost £30 each. He had tried different ways of carrying the device, including under his collar and in his pocket, but irreparable damage seemed to occur from his forgetting to remove it before his clothes went through the laundry. His suggestion to improve the device was to *“make it bigger”*. George was one of very few residents at any of the three homes who retained capacity to manage his own finances. Despite the activity tracker being funded organisationally rather than personally, this quote suggested that he was aware of the repeated expense of replacing the device.

In summary, there were numerous business and environmental influences within implementation of monitoring technologies that primarily related to the ethics of equality of access. There was a need to compromise between generic designs that might not be universally suitable, but were more affordable than bespoke designs. There may have been some challenges for care homes in managing resource distribution against individual demands. Organisational contracts with suppliers limited the scope and flexibility around product choice and ongoing maintenance. There were ongoing costs for the homes in replacing technologies that appeared to be easily damaged in the demands of daily practice. There was a sense that seeing expensive technologies as the primary source of innovative practice might be overstated or unrealistic from a business perspective.

## Discussion

This paper presents multiple-case study research exploring the implementation of monitoring technologies in three care homes for people with dementia. The findings explore the extent to which remote monitoring of care home staff, and equality of access to technologies, are seen to influence the use of monitoring technologies within routine practice. We now discuss the subsequent ethical implications.

Findings suggested that there seemed to be little overt recognition of any ethical implications of workforce monitoring upon staff members. Remote workforce monitoring was understood largely according to its potential utility to fulfil a duty of care to residents, with appeals to enhancing quality of care through guarding against poor practice (or accusations thereof). One staff member explicitly invoked the “nothing to hide” argument, which is at its most compelling in situations where the value of security is perceived to outweigh the value of privacy [44]. This argument is often predicated upon the assumption that privacy is synonymous with the concealment of specific, blatantly undesirable behaviours (and hence its appeal is perhaps understandable given the generally poor reputation of institutional care), but fails to recognise that privacy is a pluralistic concept with many different forms (e.g. spatial privacy, data and informational privacy) that may also be threatened from the accumulated monitoring of much more minor behaviours [44]. Recently, principles for use of monitoring technologies have been proposed in an attempt to provide guidance about such issues, including storage of and access to data collected by technologies [45], but the translation of these into implemented practice appears to be some way off. In our study, staff who appeared to hold negative perceptions of workforce monitoring seemed to highlight a general unease that speaks to a more cumulative form of surveillance, manifest in rumours about management motivation for technological use. However, any concerns about remote monitoring raised by management appeared to be about the extent to which data captured by monitoring technologies could constitute ‘evidence’ of appropriate care, and thus seemed to be made from a utility perspective rather than from ethical considerations of staff discomfort about cumulative surveillance.

Ethical objections to the use of technologies that could monitor the workforce were primarily made in relation to the potential impact upon residents rather than upon staff. In rejecting the use of video technology, Conifer Gardens’ managers appealed to a culture of openness which valued the personal judgements of their staff about whether the care they were providing would be good enough for their own families. This is a common argument, which in the UK explicitly guides the principles of social care inspection and regulation [32]. It would seem that more work is required to understand how technological monitoring (particularly via video) may be seen as complementary rather than mutually exclusive to staff members’ personal judgments about the quality of care’. Some work to address this question has been developed recently, including the Alzheimer’s Society’s dementia-friendly technology charter [46], and Fisk’s challenge to the notion that video cameras are purely omniscient tools of authority [45]. Such work is welcome and urgently needed. That it arguably raises more questions than it answers – for example, clarification about ownership of video data (e.g. by residents or families) may then give rise to a conflict of interest regarding its permitted use (e.g. to protect staff from false accusations) – highlights the ongoing difficulties in navigating this topic. There may be a need to clarify the roles that monitoring technologies can play in addressing concerns about abuse and neglect; these two concepts are often conflated but the former arises from deliberate maliciousness whilst the latter arises from passive ignorance, and thus there may be different implementation connotations [47]. In the UK, media depictions of care homes as rife with abuse have conflated these issues and have created a stereotype that has substantially undermined public confidence in the sector [48]; it is telling that the management at one of the homes in our study explicitly highlighted this stereotype as influential in their decision to implement monitoring technologies, and management at another home alluded to it.

Remote technological monitoring, especially covert in nature, may have implications for workforce retention and recruitment, which are rarely considered. In the present study, the covert installation of the door monitors at Heather Grove seemed to have been carried out with the assumption that staff had no choice but to accept the technology, and seemed to have led to feelings of mistrust between staff and management. It therefore seems pertinent to question the potential impacts of (covert) technological monitoring upon feelings of trust, respect, the apparent continuing problem of a blame culture within the health and social care system [49], and the attactiveness of the sector as a career option (29). Such questions are particualrly relevant at a time when there is ongoing confusion and uncertainty about the impact upon the health and social care workforce from the UK’s impending withdrawal from the European Union [50]. These questions require further scrutiny; use of technologies for workforce monitoring is likely to bring challenge from trade unions who are concerned with the legal and ethical impacts upon their members’ rights at work [51]. The covert use of the door monitors at Heather Grove was facilitated by their small size (around 25cm^2^ and very thin); increasing sophistication of ever-smaller and ever-cheaper technologies will continue to support covert remote workplace monitoring [6, 28] and hence the ethical and legal implications of such practice require urgent overt discussion.

Technological design and cost were also cited as important for equality of access to technologies. Design variation is important for the acceptance of assistive technologies by older adults [52], and for functional performance [53, 54], but is generally more expensive than generic design [55]. In the present study, the care homes met the cost of all the monitoring technologies, and needed to compromise between generic design and affordability of variation. They also had to meet ongoing costs in replacing technologies damaged in the demands of daily practice, or through maintenance agreements with suppliers. Participants expressed preferences for greater choices of products, but organisational policies and/or contracts with suppliers limited scope and flexibility. A recent study of technologies to support people with long-term conditions in their own homes called for designers to move away from a ‘walled garden’ model of exclusivity, and embrace interoperability to allow creative combination of devices and platforms to suit individual needs [56]. The present study shows that these findings appear to be similarly applicable in the care home setting. Findings also suggested that it might be overstated and unrealistic to view expensive monitoring technologies as the pinnacle of innovative practice in care homes. This challenges the utopian axiom of technological progress that pervades global health care policy, namely, that technological innovation is a self-evident value with little critique or analysis of financial cost [57, 58], and serves to reinforce recognition that the uptake of technologies into practice is context-dependent.

At present, it is possible that nuanced ethical considerations regarding equality of access to monitoring technologies in the care of older adults, particularly those who are resident in long-term care facilities, is obscured by these adults’ membership of a generation that shows by far the lowest levels of use and familiarity with contemporary technologies. In the UK, fewer than one in five adults over the age of 65 owns a smartphone, compared to around one in two aged 55–64 and almost all under-55 s [59]. There are also signs that older adults who have been users of technologies disengage with technologies, but little is known about this phenomenon [60]. It seems reasonable to suggest that considerations of equality of access to novel monitoring technologies is not prioritised when thinking about care for a generation of people who are the least likely to have technological familiarity and expectations. However, future generations of long-term care residents will have greater levels of technological familiarity and expectation; likely bringing with them personal technologies such as smartphones, wearable health technologies, and accompanying monitoring apps. There is a danger that if long-term care facilities are unprepared and ill-equipped to support new residents to access personal technologies, equality of access may become more of a pertinent issue.

### Study strengths and limitations

The main strength of this study is the application of a rigorous case study approach using an iterative and reflexive combination of research methods. As outlined above, case study methodology is well-suited to research exploring implementation of complex interventions in specific contexts. Our approach facilitated deep exploration of three distinct cases of technological implementation in the distinct contexts of three dementia-specific care homes, drawing upon the perspectives of staff members, resident and relatives and consulting documentation pertaining to the use of monitoring technologies. Our within-case analysis and cross-case synthesis has helped us to move the ethical debate around monitoring technologies beyond the apparently irreconcilable conflict of safety and autonomy, to include consideration of the impacts of workforce monitoring and equality of access to technologies upon implementation. Case study research leads to findings that are generalisable to theoretical propositions rather than at the statistical level [36]. Our findings exploring the extent to which remote monitoring of care home staff, and equality of access to technologies, are seen to influence the use of monitoring technologies within routine practice would appear to be theoretically transferrable at least to other long-term care contexts, and perhaps beyond to wider contexts.

The main limitation of our study is the selection of three care homes all located in urban settings in the same broad region of one country. It is possible that homes in rural settings or in different regions or jurisdictions may have engendered somewhat different findings. Similarly, we acknowledge the demographic homogeneity of participants, who were mainly female and White British. We also acknowledge the challenges of directly involving residents with cognitive impairment, but we followed the best available model to guide recruitment and involvement at the time the fieldwork was conducted. As highlighted in the introduction, the international debate regarding workforce monitoring and equality of access in long-term care settings is relatively under-developed, and therefore our paper is a contribution from the context of the English care sector to what we hope will be a growing field of empirical research.

## Conclusion

Remote technological monitoring of the workforce may be perceived as a potentially useful mechanism to enhance resident care and safety, either for management to hold staff to account, or to defend care homes more widely against accusations of neglectful practice. Ethical objections to remote workforce monitoring may centre on perceived utility, or the collateral impact upon resident privacy. There appears to be little consideration of the potential impacts upon staff members themselves, which may be pertinent for workforce recruitment and retention. The present study has highlighted that the power of a normative blame culture within social care may emphasise the appeal of technologies to monitor the workforce, to the detriment of considering other benefits or challenges from their use. In future practice, it may be beneficial for care homes to attempt to generate a deeper understanding of a range of benefits and challenges from using monitoring technologies, and their compatibility with ethical values and priorities of care. This may come from higher levels of involvement of staff, relatives and residents in discussions and decision-making regarding the use of monitoring technologies, and clear and regular evaluation of the impact of monitoring technologies. Future research into the perceptions of all stakeholders about the potential for remote monitoring of the workforce, and the legal implications of this practice in different jurisdictions, would be beneficial.

Generic designs of technologies might not be suitable for all residents, but ensuring variety of design is financially challenging for organisations that run care homes. Organisational policies and/or contracts with suppliers are likely to restrict the scope and flexibility of product choice and maintenance. It is possible that considerations of equitable access are not deemed a priority due to the current generation of long-term care residents’ technological familiarity and expectation. For future practice, it is recommended that care homes develop deeper knowledge and understandings of the customisability of technologies to suit their environment, and the financial implications of choosing technologies, including contractual arrangements with suppliers. It might be overstated and unrealistic for care homes to view expensive technologies as the pinnacle of innovative practice; rather, technologies could be used as part of innovative practice that is sensitive to the financial considerations of the context in which they are implemented. Future research into innovative product design at lower cost would be beneficial, as would a focus on the ways in which technologies are marketed to the care home sector.

## Additional file

Additional file 1: Example interview prompts (DOCX 13 kb)

## Abbreviations

NHS: National Health Service
