# Supplementary material for: Moving beyond ‘safety’ versus ‘autonomy’: a qualitative exploration of the ethics of using monitoring technologies in long-term dementia care
Source: BMC Geriatr. 2019 May 24;19:145. doi: 10.1186/s12877-019-1155-6 (PMC6534927; doi:10.1186/s12877-019-1155-6)
Supplement: Supplementary file 1 — Example interview prompts (DOCX 13 kb) [file 12877_2019_1155_MOESM1_ESM.docx]

**Additional file 1. Example interview prompts**

• Can you describe what you thought [name of technology] was?

• Did you get a sense that [name of technology] was different to anything you had done previously in [name of organisation]?

• Did you believe there was a place for [name of technology] within [name of organisation]?

• What did you think that your role might be in using [name of technology]?

• How did you think that [name of technology] would affect you?

• What was the general feeling amongst people at that time regarding [name of technology]?

• Can you describe how people felt when they learned about [name of technology]?

• Who did you think would benefit from [name of technology]? And how?

• What do you believe in most about caring for people with dementia and how do you feel about the use of [name of technology] in relation to this?

• Does [name of organisation] have particular values that it promotes? How do you feel about the use of [name of technology] in relation to these values?

• What role did you see for [name of technology] within [name of organisation]?

• Can you describe how [name of technology] was put into practice?

• Whose involvement do you see as being necessary for [name of technology] to have high impact?

• Was anyone in charge of [name of technology]?

• Did your role change as use of [name of technology] got underway?

• Whose involvement do you see as necessary for using [name of technology]?

• How did you feel about getting involved in using [name of technology]?

• How do you find using [name of technology]? Is there anything easy or anything difficult about using it?

• Does [name of technology] make your life easier?

• Can you describe how you use [name of technology] on a day to day basis?

• Does everybody know what their responsibilities are around [name of technology]?

• Do you trust [name of technology]?

• Have you had any problems with faulty technology?

• Do you feel that [name of organisation] supports the use of [name of technology]?

• Tell me about the information and training you have received around [name of technology]

• What do you think about [name of technology] now that you have been involved with it?

• Can you see what impact [name of technology] is having?

• Describe for me what you think is the impact of [name of technology]?

• Do you feel that people generally think that [name of technology] is having this impact?

• How do you evaluate [name of technology]?

• Do you talk about [name of technology]?

• Can you tell me about team meetings and any discussions regarding residents’ care?

• Has anything changed because of [name of technology]?

• Is there anything that you would change about [name of technology]?

• Is there anything that you would change about the way that [name of technology] is being used?
